# Biosynthesized Gold Nanoparticles from *Eruca sativa* Mill. Leaf Extract Exhibit In Vivo Biocompatibility, Antimicrobial, and Antioxidant Activities

**DOI:** 10.3390/antibiotics14080776

**Published:** 2025-07-31

**Authors:** Abdullah Muhsin Hazbar, Abdulkadir Mohammed Noori Jassim, Mustafa Taha Mohammed, Younis Baqi

**Affiliations:** 1 Department of Chemistry, College of Science, Mustansiriyah University, Baghdad P.O. Box 14022, Iraq; abdullah.m@uomustansiriyah.edu.iq (A.M.H.); kadirchem@uomustansiriyah.edu.iq (A.M.N.J.); dr.mustafa@uomustansiriyah.edu.iq (M.T.M.); 2 Department of Chemistry, College of Science, Sultan Qaboos University, Muscat 123, Oman

**Keywords:** antimicrobial, antioxidant, biocompatibility, biosynthesis, *Eruca sativa* Mill., gold nanoparticles

## Abstract

**Background/Objectives:** Antimicrobial resistance (AMR) is a health related threat world-wide. Biosynthesized gold nanoparticles (AuNPs) using plant extracts have been reported to exhibit certain biological activity. This study aimed to biosynthesize AuNPs using an aqueous extract of *Eruca sativa* leaves and to evaluate their biocompatibility, antimicrobial activity, and antioxidant properties. **Methods:** AuNPs were biosynthesized using an aqueous extract of *Eruca sativa* leaves. Their biocompatibility was evaluated through hemolytic activity and assessments of hepatic and renal functions in rats. AuNPs were biologically evaluated as antimicrobial and antioxidant agents. **Results:** The AuNPs exhibited particle sizes of 27.78 nm (XRD) and 69.41 nm (AFM). Hemolysis assays on red blood cells revealed negligible hemolytic activity (<1%). Hepatic enzyme levels, including alanine aminotransferase (ALT), aspartate aminotransferase (AST), alkaline phosphatase (ALP), and lactate dehydrogenase (LDH) were studied. ALT, AST, and ALP levels showed no significant changes compared to the negative control. However, LDH levels were elevated at higher concentration (52.8 µg/mL), while the lower concentration (26.4 µg/mL) appeared to be safer. Renal biomarkers, urea and creatinine, showed no significant changes at either concentration, indicating minimal nephrotoxicity. The antimicrobial activity of AuNPs, plant extract, and gold salt was tested against five microorganisms: two Gram-positive bacteria (*Staphylococcus aureus*, *Streptococcus pneumoniae*), two Gram-negative bacteria (*Escherichia coli*, *Pseudomonas aeruginosa*), and a fungal strain (*Candida albicans*). The AuNPs exhibited minimum inhibition concentrations (MICs) of 13.2 µg/mL against *S. aureus* and *S. pneumoniae*, 26.4 µg/mL against *E. coli* and *C. albicans*, and 39.6 µg/mL against *P. aeruginosa*, suggesting selectivity towards Gram-positive bacteria. Furthermore, the AuNPs demonstrated strong antioxidant activity, surpassing that of vitamin C. **Conclusions:** The biosynthesized AuNPs exhibited promising biocompatibility, selective antimicrobial properties, and potent antioxidant activity, supporting their potential application in combating the AMR.

## 1. Introduction

Antimicrobial resistance (AMR) is one of the most serious threats for public health worldwide, which occur due to frequent use or misuse of clinical antimicrobial drugs [1]. As a result, microorganisms, including bacteria, fungi, parasites, and viruses, develop a survival mechanism against known antimicrobial drugs, which leads to rendering these drugs ineffective [2]. Antioxidant activity of a product refers to its ability to reduce, inhibit, or prevent oxidation reactions through their ability to terminate free radicals, the presence of free radicals causing oxidation, which often lead to cell and tissue damage [3]. Therefore, there is always a need to develop new antimicrobial agents with certain antioxidant activity to combat the AMR.

Nanomaterials science is a fast-advancing field that represents several components based on the shape and dimensions of these materials, such as nanoparticles, nanowires, nanotubes, and nanosheets [4]. These nanomaterials exhibit very interesting physical, chemical, and biological applications due to their large surface area, resulting from their extremely small particle size within small volume quantity; typically these particles’ sizes range from 1 to 100 nm [5]. Over the past decades, many nanomaterials have been developed for diverse applications, in particular energy storage [6], catalysis [7], and pharmaceutical applications [8]. Biosynthesized silver nanoparticles (AgNPs) have recently been reported as promising antimicrobial agents [9]. In addition to that, nannoplankton (nannofossils) have been identified in sedimentary successions from the Jurassic period [10]. Among these nanomaterials, gold-containing nanoparticles (AuNPs) are found to be one of the most stable nanomaterials with many applications, including drug delivery [11], photothermal therapy and immunotherapy [12], as well as cancer therapy [13,14,15]. However, these AuNPs have been reported to exhibit some oxidative damage to cell lines and tissues used in vitro and in vivo, respectively. It has been concluded that liver and kidney functions are the most affected organs by the applications of AuNPs [16]. It is well documented in the literature that a greener approach such as applying biosynthetic strategies to access nanoparticles and utilizing plant extract reduces the level of toxicity present in the nanoparticles [17,18], including AuNPs [19]. This is probably due to the presence of phytochemicals in plant extracts, which are known for their nutritional value and therapeutic properties [20,21,22,23,24].

Rocket (*Eruca sativa* Mill.) is an edible plant belonging to the Brassicaceae family. This species is native to the Mediterranean region and to China through the Arabian Peninsula [25]. Phytochemical extracts from rocket have been reported to show several biological activities, including cardiovascular [26], male reproductive health [27], and antioxidants [28]. Polyphenolic compounds, including flavonoids, are the principal phytochemicals found in several tissues of rocket that enhance its antioxidant activities [29]. Thus, combining AuNPs with *Eruca sativa* extract may enhance antimicrobial effectiveness while minimizing toxicity.

In the present study, novel AuNPs were biosynthesized using a water extract obtained from the leaves of the *Eruca sativa* plant, and their biocompatibility properties were assessed using hemolytic effect on red blood cells and hepatic enzyme levels, such as alanine aminotransferase (ALT), aspartate aminotransferase (AST), alkaline phosphatase (ALP), and lactate dehydrogenase (LDH). In kidney functions, two biomarker levels were studied, these are urea and creatinine. Furthermore, the antioxidant properties were measured, and the antimicrobial activity was investigated on five different microorganisms, namely *Staphylococcus aureus* and *Streptococcus pneumoniae* (Gram-positive), *Escherichia coli* and *Pseudomonas aeruginosa* (Gram-negative), and *Candida albicans* (fungi).

## 2. Results and Discussion

### 2.1. Preparation of the Eruca sativa Leaves Extract

The *Eruca sativa* leaves aqueous extract was prepared using fresh plant leaves and heated to 60 °C to facilitate the release of bioactive compounds, enhance extraction efficiency, and increase solubility. Heating beyond 70–80 °C may lead to the degradation of these active constituents. The resulting extract exhibited a green color, primarily due to the presence of chlorophyll pigments naturally found in the plant leaves. It is recommended to store the plant extract at 4 °C and to utilize it within 24 h. We noticed that extended storage beyond this period resulted in microbial contamination and subsequent spoilage.

### 2.2. Preparation of the Gold Stock Solution and Its Dilution

**Figure 1:**
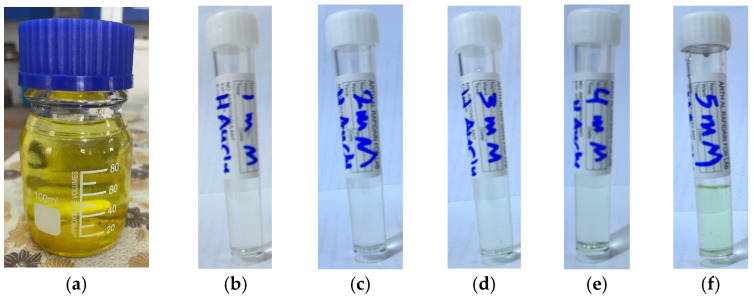
Gold salt stock and its diluted solutions: (**a**) 25 mM; (**b**) 1 mM; (**c**) 2 mM; (**d**) 3 mM; (**e**) 4 mM; (**f**) 5 mM.

Tetrachloroauric(III) acid trihydrate (HAuCl_4_·3H_2_O) is a yellow crystalline compound and fully dissolves in water producing a yellow solution (Figure 1a). The solution exhibited clear homogeneity with no visible precipitation or impurities, indicating complete dissolution of the gold salt in the aqueous medium. This step is essential to ensure the success of the subsequent reduction reaction and the uniform and stable formation of gold nanoparticles. The prepared stock solution was diluted to prepare five different concentrations (1, 2, 3, 4, and 5 mM), Figure 1b–f.

### 2.3. Green Synthesis of the AuNPs Using Eruca sativa Leaves Extract

**Figure 2:**
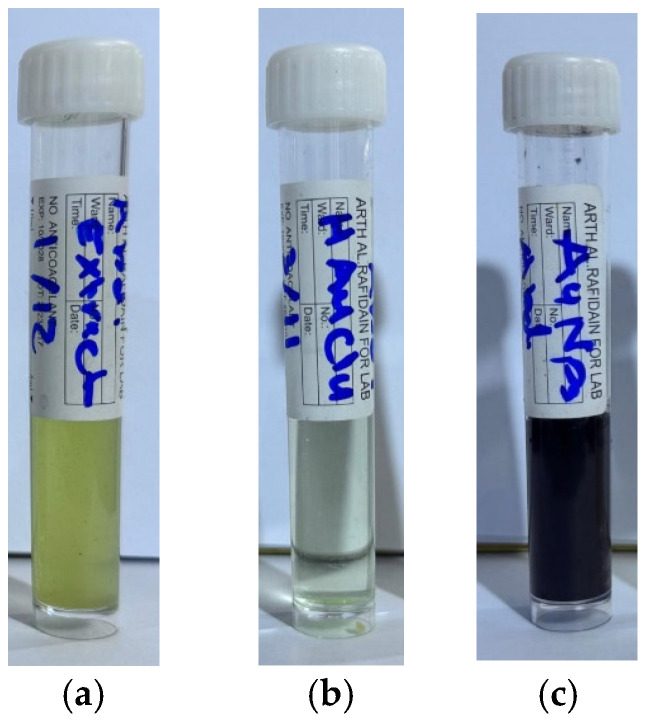
Synthesis of AuNPs: (**a**) *Eruca sativa* extract; (**b**) Gold solution [5 mM]; (**c**) AuNPs.

In the synthesis of gold nanoparticles, the plant extract (Figure 2a) was added gradually to the gold salt solution (Figure 2b) to ensure controlled nucleation and uniform particle formation. The reaction is typically carried out under elevated temperatures, which play an important role in accelerating the reduction process, promoting nanoparticle formation, and improving size distribution and stability of the resulting gold nanoparticles. The color change, typically from pale yellow to violet, serves as a visual indicator of successful nanoparticle synthesis due to the surface plasmon resonance (SPR) phenomenon associated with gold nanoparticles [30], see Figure 2c.

The preparation of AuNPs was followed by its color, which ranged from light pink to violet when using 1–4 mM concentration. This indicates instability of the nanoparticles at these concentrations. In addition, the required reaction temperature varied during preparation ranging from 75 to 90 °C. Moreover, the required volume of gold salt solution was inconsistent (6–10 mL), while the usual standard volume should be consistent. All inconsistencies in these parameters reflect the instability in the synthesized AuNPs. In contrast, using the 5 mM concentration resulted in consistent conditions for the preparation of AuNPs in terms of color, temperature, and solution volume, indicating the suitability of this concentration for the preparation of stable AuNPs. The stability of the biosynthesized AuNPs was monitored over time. The violet color remained stable for several months. Additionally, the antioxidant activity was tested twice, with a seven-week interval using the same AuNPs sample, and both tests showed similar antioxidant activity.

Using a plant extract, such as the leaves of the *Eruca sativa* plant, can improve the AuNPs with respect to several parameters, including the reduction of Au(III) ions, stabilization of the synthesized nanostructures, and the addition of some biological effects of the phytochemicals present in the plant [31]. The concentration of the extract influences the size, shape, and regularity of the produced AuNPs. The synthesis of AuNPs may be substantially altered by adjusting the reducing properties of various plant species, which contain varying levels of active reducing agents. However, when the quantities of plant extracts increase, AuNPs often decrease in size [32]. The impact of phytochemicals that form, grow, and stabilize the crystals, along with their decreased production rate, accounts for the reduction in size. This has prompted scientists to develop synthetic strategies that provide enhanced control over size and form [33].

### 2.4. UV-Vis Spectroscopy Study

**Figure 3:**
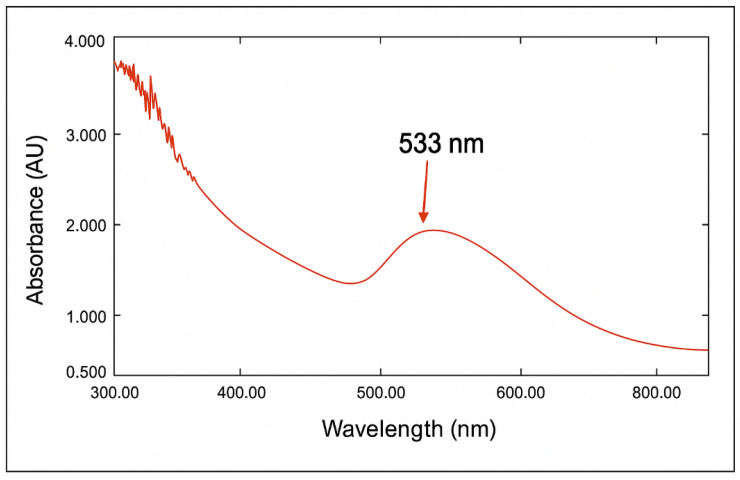
UV-Vis spectrum of the biosynthesized AuNPs using *Eruca sativa* plant extract.

The characteristics of AuNPs were determined by UV-Vis spectroscopy. The data revealed the absence of a distinct band for the *Eruca sativa* extract; however, upon the addition of the gold solution (HAuCl_4_), a broad peak emerged within the range of 530–540 nm [34]. The λ_max_ at 533 nm indicates the development of monodispersed spherical AuNPs (Figure 3), as previously reported [35], and this is confirmed by other characterizations. This reaction transpires in about 10 min, accompanied by a noticeable color shift. The color shift is a recognized occurrence in nanoparticle synthesis, especially concerning AuNPs, where the color alteration serves as a visual indicator of nanoparticle creation [32].

### 2.5. Field Emission Scanning Electron Microscopy (FESEM) Analysis

**Figure 4:**
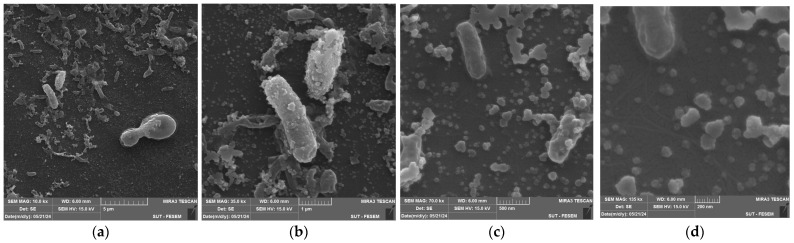
FESEM images of synthesized AuNPs at different SEM magnification: (**a**) 10.0 kx; (**b**) 35.0 kx; (**c**) 70.0 kx; (**d**) 135.0 kx.

The morphology and dimensions of the biosynthesized AuNPs were analyzed using FESEM, which shows the particle size distribution histogram of AuNPs. Pictures in Figure 4 are taken at different SEM magnifications: 10.0 kx (a), 35.0 kx (b), 70.0 kx (c), and 135.0 kx (d); these demonstrate that the generated AuNPs display several morphologies, with the spherical form being the most prevalent. The mean particle sizes were analyzed using the ImageJ 1.x program and were found to be around 37–70 nm.

### 2.6. Atomic Force Microscopy (AFM) Analysis

**Figure 5:**
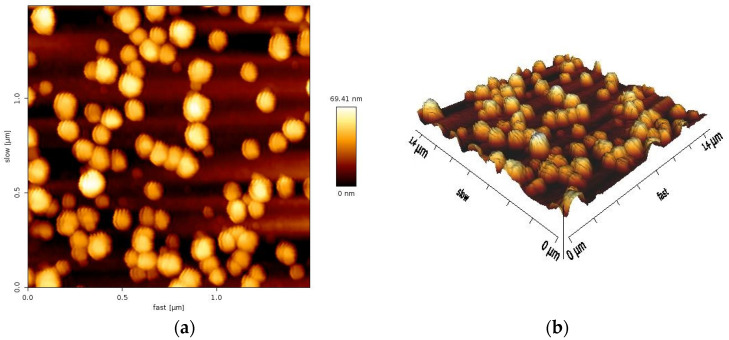
AFM images and size distributions of AuNPs synthesized: (**a**) 2D AFM image; (**b**) 3D AFM image.

The biosynthesized AuNPs were studied by AFM analysis to determine their size and shape. The origin of the surface morphology was found to be irregularly shaped particle sizes and the wide size range of AuNPs prepared using *Eruca sativa* plant extract. The particle size of the AuNPs ranged from 20 to 90 nm, with an average size of 69.41 nm. Figure 5 illustrates the 2D and 3D AFM images together with the associated size distribution of AuNPs. The AFM sample taken one month following the synthesis of AuNPs exhibited no aggregation or agglomeration, which indicates that these AuNPs exhibit very good stability. The obtained particle size is consistent with the published results from earlier investigations [36]. Other researchers reported the particle size of AuNPs with different sizes [37]. The discrepancies in particle sizes of the biosynthesized AuNPs are probably due to the utilization of different synthesis approaches and/or variations in plant species.

### 2.7. X-Ray Diffraction (XRD) Analysis

**Figure 6:**
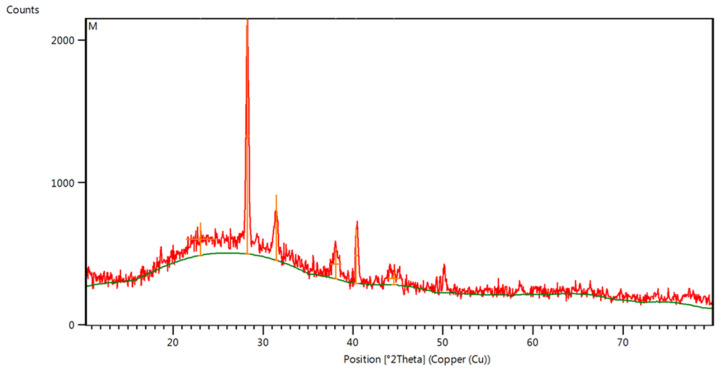
XRD pattern for the biosynthesized AuNPs.

The XRD pattern of the synthesized AuNPs is illustrated in Figure 6. The XRD peaks observed at 2θ values of 23.05°, 28.23°, 31.49°, 38.11°, 40.35°, and 44.57° correspond to the following peaks, respectively: 111, 200, 220, 311, 222, and 400. The findings suggested that the biosynthesized AuNPs exhibited a polycrystalline structure. The (200) peak exhibited significant strength, which signified the preferred orientation of the AuNPs. Additionally, peaks associated with the AuNPs identified as 111, 220, 311, 222, and 400 were observed and analyzed in relation to ICDD data. The sharp and intense diffraction peaks of AuNPs suggested that the resulting products exhibited a high degree of crystallinity. The AuNPs’ crystal size was determined using the Debye–Scherrer equation and was 27.78 nm, corresponding to the (200) peak.

### 2.8. Zeta Potential Analysis

**Figure 7:**
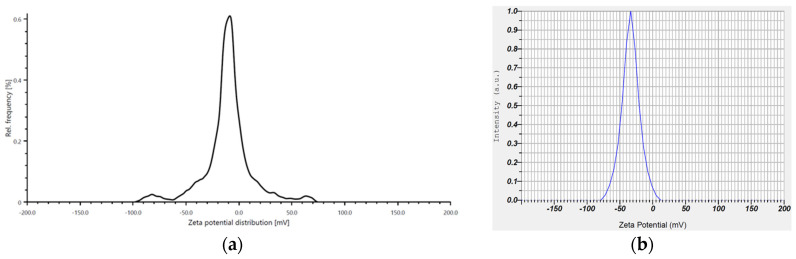
Zeta potential analysis: (**a**) *Eruca sativa* extract; (**b**) biosynthesized AuNPs.

The zeta potential analysis of the pure *Eruca sativa* extract was −11.6 mV, while for the biosynthesized AuNPs it decreased to −33.8 mV (Figure 7). As a result, the AuNPs exhibited adequate stability, with readings meeting or exceeding the necessary stable expression criteria. The generated AuNPs exhibited a consistent shape for more than a month and demonstrated no indications of aggregation or agglomeration.

### 2.9. Inductively Coupled Plasma–Mass Spectrometry (ICP-MS) Analysis

ICP-MS was used to examine the plant extract and AuNPs in order to quantify trace minerals in plants, assess the impact of AuNPs, and observe any alterations caused by the presence of AuNPs. The concentrations of various elements, including gold, in *Eruca sativa* were measured in µg/mL units. Gold nanoparticles were not detected in the plant extract but were found at a concentration of 52.8 µg/mL in the biosynthesized AuNPs sample.

### 2.10. Hemolysis Assay

The hemolytic activity of the biosynthesized AuNPs was measured and compared with a positive control (PC) and a negative control (NC). Deionized water was used as the positive control and represents complete hemolysis, with an absorbance of 0.991 AU. Saline was used as the negative control and showed an absorbance of 0.087 AU, indicating no hemolysis. All measurements were conducted at a fixed wavelength of 415 nm, which corresponds to the maximum absorbance of hemoglobin. An increase in absorbance intensity indicates greater hemoglobin release, which reflects a higher level of hemolysis [38]. The hemolytic activity results for the AuNPs and the controls are summarized in Table 1. The biosynthesized AuNPs showed an absorbance of 0.095 AU, which is very close to that of the negative control. This indicates that the AuNPs synthesized using *Eruca sativa* leaf extract do not induce significant red blood cell lysis.

The hemolysis coefficient (HC) was calculated using the equation mentioned in the experimental part and was found to be <1%, which indicates that the biosynthesized AuNPs exhibit minimal hemolytic activity and, thus, are not significantly toxic to red blood cells under the conditions tested. This hemolytic result suggests that AuNPs are biocompatible and have very low toxicity toward red blood cells and hence can be utilized in medical applications, including drug delivery or diagnosis, without causing significant damage to red blood cells.

### 2.11. Hepatic and Renal Functions

#### 2.11.1. Hepatic Function

**Figure 8:**
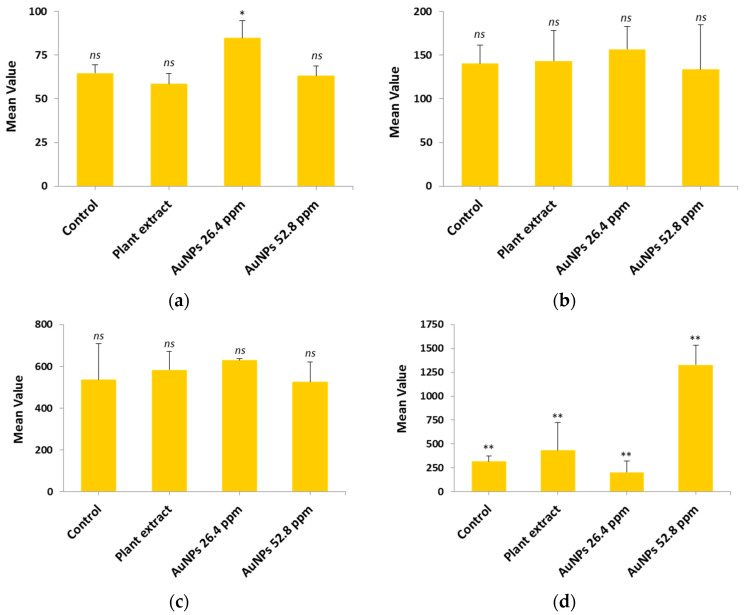
Comparative mean levels of liver function among study groups: (**a**) Alanine transaminase (ALT); (**b**) Aspartate aminotransferase (AST); (**c**) Alkaline phosphatase (ALP); (**d**) Lactate dehydrogenase (LDH). Statistical significance is indicated as follows: *p* < 0.05 (*), *p* < 0.001 (**), and *ns* (non-significant).

The hepatic function levels for the four groups (Control, Extract, AuNPs 26.4 µg/mL and AuNPs 52.8 µg/mL) were measured and are presented as mean ± SD, as shown in Table 2 and Figure 8.

The results suggest that there is no significant difference among ALT, AST, and ALP for all tested materials, including control (deionized water), plant extract, AuNPs (26.4 mM), and AuNPs (52.8 mM), except at the lower concentration of AuNPs (26.4 mM) on ALT, which increased the ALT level significantly. This is probably due to mild hepatocellular stress or damage induced by the lower concentration (26.4 mM) in vivo, resulting in elevated ALT release into the circulation [40]. On the other hand, the LDH enzyme level shows significant difference with respect to the high concentration of using AuNPs (52.8 mM), while at a lower concentration there was no toxicity observed. This may be due to the fact that LDH is a general biomarker of cellular membrane damage and cytotoxicity, which may be caused by enzyme leakage from various tissues, not only the liver [41].

#### 2.11.2. Renal Function

**Figure 9:**
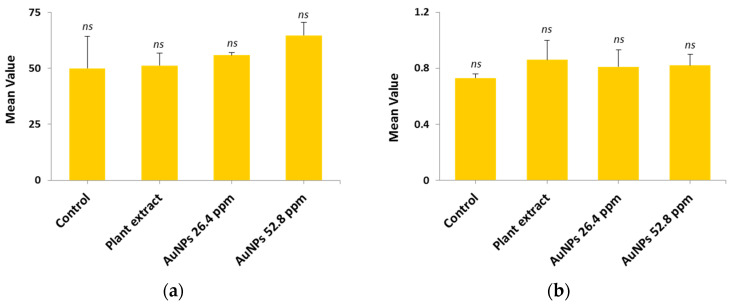
Comparative mean levels of kidney function indicator among study groups: (**a**) Urea; (**b**) Creatinine; *ns* refers to non-significant.

In order to study the renal function, two parameters (urea and creatinine) were selected based on their importance with regard to the efficacy of renal filtration and overall renal health. All groups, including the control, plant extract, AuNPs (26.4 µg/mL), and AuNPs (52.8 µg/mL) were measured and the mean ± SD are illustrated in Table 3 and Figure 9.

The results showed no significant difference (*p* > 0.05) among tested groups; this indicates there is no change in the normal urea and creatinine levels and therefore there is no impact on kidney cells.

### 2.12. Minimum Inhibitory Concentration (MIC)

**Figure 10:**
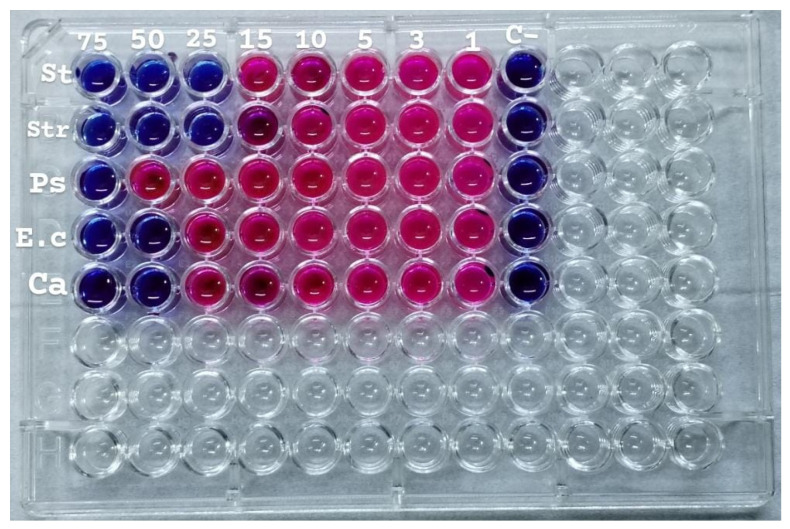
The microtiter plate infected with double serial dilutions of the test chemicals and resazurin dye. 1–75: Percent concentration of AuNPs (52.8 µg/mL); C−: Negative Control; St: *S. aureus*, Str: *S. pneumoniae*; Ps: *P. aeruginosa*; E.c: *E. coli*; Ca: *C. albicans*.

The AuNPs were evaluated in vitro against five microbial isolates: *S. aureus* and *S. pneumoniae* (Gram-positive), *P. aeruginosa* and *E. coli* (Gram-negative), and *C. albicans* (fungus). The MIC was determined by solution color change on the plate: pink/red wells mean no microbial growth inhibition observed, while the blue/purple color wells represent lack of microbial growth, which means inhibition of microorganism growth. The MIC results are as follows: *S. aureus* and *S. pneumoniae* (13.2 µg/mL), *P. aeruginosa* (39.6 µg/mL), *E. coli* and *C. albicans* (26.4 µg/mL), see Figure 10.

### 2.13. Antimicrobial Activity

The antimicrobial activity of the biosynthesized AuNPs, plant extract, and gold salt (HAuCl_4_) using the well diffusion method was conducted on five microorganisms: two Gram-positive (*S. aureus*, *S. pneumonia*), two Gram-negative (*P. aeruginosa*, *E. coli*), and one fungus (*C. albicans*). The minimum inhibitory concentration MIC results obtained in Section 2.12 were utilized to measure the antimicrobial activity, see Table 4.

The values presented in Table 4 show zones of inhibition (measured in millimeter), demonstrating the efficacy of each drug in inhibiting microbial growth. Elevated readings indicated enhanced efficacy.

The zones of inhibition ranged from 35 mm to 40 mm, with selectivity against *S. aureus*, *S. pneumoniae*, and *C. albicans* (40 mm for each). The selectivity of AuNPs on Gram-positive bacteria vs. Gram-negative bacteria is probably due to the fact that nanoparticles may penetrate the thicker cell membrane of Gram-positive bacteria and interact with interior cellular components. In contrast, in Gram-negative bacteria, the cell wall is shielded by a bilayer membrane, which might influence the permeability of the AuNPs, resulting in a less significant impact [42]. The plant extract showed a moderate activity, with inhibition zones ranging from 34 to 37 mm. HAuCl_4_ (gold salt) showed the least effectiveness of the tested products with zones of inhibition in the range of 28–30 mm. The results revealed that AuNPs were the most effective antibacterial agent, likely due to their high reactivity and ability to penetrate microbial cells due to their extremely small size (70 nm).

With regard to the mechanism of action, recent studies suggest that gold nanoparticles (AuNPs) exhibit antibacterial activity through multiple integrated mechanisms. For example, AuNPs can interact with bacterial cell walls, disrupting membrane integrity and increasing permeability, leading to leakage of cellular contents and eventual cell death. This mechanism is comparable to the inhibitory effect observed with silver nanoparticles [43]. In another study, AuNPs are capable of inducing the generation of reactive oxygen species (ROS), which cause oxidative damage to cellular proteins, lipids, and DNA. This oxidative stress leads to severe impairment of bacterial cell function [44]. Moreover, AuNPs may bind to intracellular proteins and nucleic acids, interfering with transcription and translation processes, thereby inhibiting bacterial replication. This mechanism is supported by findings from studies on organo-ligand-based nanomaterials with confirmed antibacterial efficacy [45]. These findings suggest that AuNPs probably act through a multifaceted approach, targeting bacterial cells through physical disruption and biochemical interference, leading to effective antimicrobial action.

### 2.14. DPPH Free Radical Scavenging Activity

**Figure 11:**
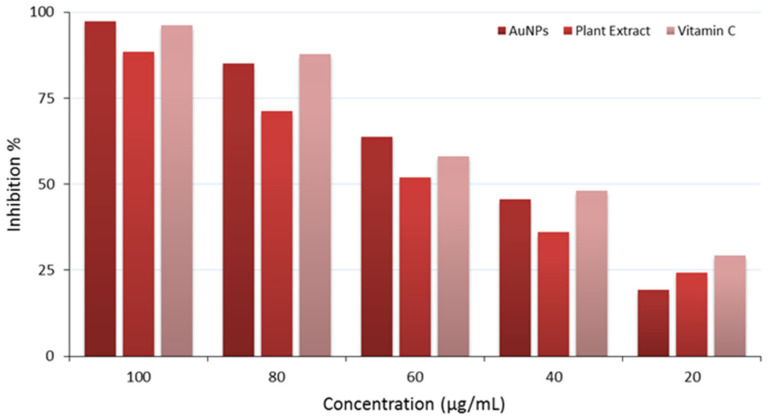
Antioxidant properties of the AuNPs (dark red color), plant extract (red color), and vitamin C (light red color).

The antioxidant activity was conducted on the biosynthesized AuNPs, the plant extract, and vitamin C; results are illustrated in Table 5 and Figure 11.

The findings assess the antioxidant activity of three distinct substances: AuNPs, plant extract, and vitamin C, evaluated at varied doses (100, 80, 60, 40, and 20 µg/mL). The inhibition percentages were quantified to assess their efficacy in neutralizing reactive oxygen species (ROS), which are significant contributors to oxidative stress and cellular damage. All three tested materials (AuNPs, plant extract, and vitamin C) showed a concentration-dependent manner. AuNPs showed almost full percent inhibition (97.2%) at 100 µg/mL. It shows slightly stronger antioxidant activity compared to the standard (vitamin C). Interestingly, the plant extract showed notable antioxidant activity, demonstrating an inhibition percentage of around 88.5% at 100 µg/mL. This antioxidant activity is probably due to the presence of phytochemicals in the plant extract. The IC_50_ values for AuNPs, *Eruca sativa* extract, and vitamin C are 47.55, 54.55, and 48.04 µg/mL, respectively. The novel gold nanoparticles showed a slightly lower IC_50_ than vitamin C, indicating their ability to suppress oxidation.

## 3. Materials and Methods

### 3.1. General

All chemicals were used as purchased from respective companies: Tetrachloroauric(III) acid trihydrate from Merck KGaA, Darmstadt, Germany; 1,1-diphenyl-2-picrylhydrazyl (DPPH), vitamin C, copper sulfate, ferric chloride, methanol, potassium mercuric iodide, potassium iodide, resazurin dye from Sigma-Aldrich, Darmstadt, Germany. All stock solutions and further dilutions were prepared using deionized water. Healthy Wistar rats (male) were obtained from the Biotechnology Research Center, Al-Nahrain University, Baghdad, Iraq. Atomic Force Microscope (AFM), Model AA3000, Angstrom Advance Inc., Stoughton, MA, USA; Centrifuge (Digital), Rotina, Tuttlingen, Germany; FE-SEM, FESEM MIRA3 TESCAN instrument, Cranfield, UK; FT-IR-shimadzu-8400S spectrophotometer, Shimadzu-8400S, Tokyo, Japan; UV-Vis Spectrometer, PG-Instruments Limited, T80, Lutterworth, UK); Cobas c111 Automated Analyzer (Roche, Mannheim, Germany); XRD, X-RD equipment X-Pert Pro, Georgia, Zeta Plus instrument, Brookhaven Instruments Corp., Nashua, NH, USA; and ICP-MS, X-Series II ICP-MS, Thermo Fisher Scientific Inc., Waltham, MA, USA.

### 3.2. Preparation of the Eruca sativa Mill. Leaves Extract

The rocket (*Eruca sativa* Mill.) plant was freshly collected near the city of Baqubah, Diyala, Iraq. It was identified by a plant taxonomist, Prof. Dr. Khazal Dabaa Wadi, Department of Biology, College of Science, Diyala University, Iraq. The leaves were taken from the plant, washed three times with tap water and finally with distilled water to remove any mud or dirt. The leaves were chopped into small pieces around 1 cm^2^, using sterilized scissors. The small pieces of rocket (20 g) were mixed with distilled water (100 mL) and heated using hot plate at 60 °C for one hour. The mixture was then allowed to cool down to room temperature. The solution was then filtered through Whatman filter paper No. 1. The filtrate was collected and placed in a sealed glass bottle and stored at 4 °C until it was used for the preparation of AuNPs.

### 3.3. Preparation of the Gold Stock Solution and Its Dilution

The gold solution was prepared by dissolving tetrachloroauric(III) acid trihydrate (1 g) in deionized water (100 mL) resulting in a 25 mM concentration (Figure 1a). From this stock solution, 1, 2, 3, 4, and 5 mM solutions were prepared, corresponding to Figure 1b–f, respectively, and utilized for the green synthesis of AuNPs.

### 3.4. Green Synthesis of AuNPs

*Eruca sativa* plant extract (10 mL) was gradually added to the gold salt solutions at varying concentrations (1, 2, 3, 4, and 5 mM) over 15 min at 60 °C. The 5 mM concentration gave the most stable and reproducible AuNPs (Figure 2). The formation of AuNPs with the *Eruca sativa* extract was followed by the change in the reaction mixture color from light yellow to violet, which serves as a preliminary indicator for the synthesis of AuNPs [46].

### 3.5. Characterization of the Biosynthesized Gold Nanoparticles (AuNPs)

#### 3.5.1. Ultraviolet–Visible (UV–Vis) Spectroscopy

The UV-Vis spectrum was obtained using a spectrophotometer. At regular intervals, spectroscopic assays assessed the reduction of AuNPs. The wavelength range was 300 to 800 nm. Three milliliters of the sample were put into a UV cuvette and subsequently assessed at ambient temperature. UV-Vis spectroscopy is a useful tool for the characterization of nanoparticles [47].

#### 3.5.2. The Field Emission Scanning Electron Microscopy (FE-SEM) Analysis

The morphology of the prepared AuNPs was examined using FE-SEM instrument. This analysis provides high resolution pictures of the surface of the nanoparticles, which makes this technique useful for the characterization of nanoparticle size distribution [48].

#### 3.5.3. The Atomic Force Microscopy (AFM) Analysis

The samples were analyzed using an AFM to determine the precise particle size and quantify the nanosize effect. The method of evaporating the droplet was used to create AFM suspension samples of nanoparticle fluid. The drop was placed on a glass cover slide (2 × 6 cm^2^) and then dried before imaging, either by leaving it overnight in a dust-free environment or by using an oven/heater at a low temperature to expedite the drying process [49].

#### 3.5.4. X-Ray Diffraction (XRD) Analysis

The crystal-phase structure and crystallite size of the AuNPs were determined using XRD equipment (X’Pert Pro), which operated within a scanning range of 2θ from 10° to 80°, at a wavelength of λ = 1.5418 Å using CuKα radiation, operating at 40 kV and 30 mA. The crystallite size of the AuNPs was calculated using the Debye–Scherrer equation:

D = Kλ/(β cos θ): where D is the crystallite size, λ is the X-ray wavelength (0.15406 nm for CuKα), K is the shape factor (commonly 0.89), β is the full width at half maximum (FWHM) of the diffraction peak in radians, and θ is the Bragg diffraction angle.

#### 3.5.5. Zeta Potential Analysis

The zeta potential experiment was conducted to characterize both the biosynthesized gold nanoparticles (AuNPs) and the *Eruca sativa* extract. Zeta potential was measured using light dispersion techniques with the Zeta Plus instrument. The data included five key parameters. The size measurements of the nanoparticles were assessed using Electrophoretic Light Scattering (ELS) and Dynamic Light Scattering (DLS). Surface characterization and the calculation of surface charge of the nanomaterials were accomplished through zeta potential analysis.

#### 3.5.6. Inductively Coupled Plasma–Mass Spectrometry (ICP-MS) Analysis

An ICP-MS instrument was utilized to determine the total elemental concentrations in colloidal solutions at sub-parts-per-trillion levels. In addition to elemental analysis, modern ICP-MS methodologies have expanded its applicability to include the size characterization of nanoparticles. This includes the use of single-particle ICP-MS (spICP-MS) to examine the size distribution of nanoparticles, including colloidal gold.

### 3.6. Ethical Approval

This research was approved by the Scientific and Ethical Committee Ref.: BCSMU/0524/0003C on 1 May 2024, Department of Chemistry, College of Science, Mustansiriyah University. It meets the Three Rs requirements (replacement, reduction, and refinement).

### 3.7. Hemolytic Activity

In rat whole blood, the hemolytic ability of AuNPs was investigated as previously described [50]. In brief, 0.5 mL of the AuNPs sample was incubated with a corresponding volume of blood, obtained from rats, for 24 h at 37 °C in a biological thermostat. Blank samples were prepared; deionized water served as the positive controls (PC) and saline as the negative controls (NC). Following incubation, the samples were vortexed for 5 min. The mixture was analyzed using spectrophotometer at a wavelength of 415 nm, which is similar to the absorption band of oxyhemoglobin, hemolytic activity was assessed using the hemolysis coefficient (HC). The following formula was used to compute hemolytic activity:

HC = (O − NC)/(PC − NC) × 52.8 µg/mL

O represents the optical density of the sample, NC denotes the negative control (0% hemolysis of the blank sample), and PC signifies the positive control (52.8 µg/mL hemolysis of the blank sample) [51]. The AuNPs concentration was obtained using ICP-MS.

### 3.8. Animals and Experimental Design

The experimental animals, male Wistar rats (6–8 weeks old, weighing 200–250 g), were housed in the animal facility of the Biotechnology Research Center, Al-Nahrain University, Baghdad, Iraq, and were handled by a specialized team at the center. Sixteen rats were randomly distributed into four groups of four rats each. Group 1 received deionized water and served as the control; group 2 received the extract at 50 mg/kg body weight orally once; group 3 was administered the concentrated dose of AuNPs (52.8 µg/mL); and group 4 received diluted AuNPs (26.4 µg/mL). All test animals received oral gastric gavage once a day with varying materials (deionized water, plant extract, and AuNPs) and each rat was continuously monitored every day for 28 days post-administration [52].

### 3.9. Hepatic and Renal Function Analysis

#### 3.9.1. Blood Samples Collection

Upon completion of the treatments, the rats were anesthetized with diethyl ether, and blood samples were procured by heart puncture into gel/clot activator tubes for 30 min to expedite clotting, followed by centrifugation at 3000 rpm for 10 min to isolate serum. Blood specimens for hematological analysis were obtained in an EDTA tube [53].

#### 3.9.2. Serum Analysis of Hepatic and Renal Function

The following liver function parameters were assessed by measuring alanine aminotransferase (ALT), aspartate aminotransferase (AST), alkaline phosphatase (ALP), and lactate dehydrogenase (LDH). Renal functions were assessed by the measuring of urea and creatinine.

### 3.10. Antimicrobial Activity

The agar well diffusion method was utilized to evaluate the antibacterial activity of AuNPs at their MIC. The antibacterial activity of *Eruca sativa* extract, AuNPs, and HAuCl_4_ solution was tested against *S. aureus* and *S. pneumonia* (Gram-positive), *E. coli* and *P. Aeruginosa* (Gram-negative), and *C. albicans* (fungus). Each bacterial isolate was cultivated in nutrient broth and incubated at 37 °C for 18 to 24 h. Following the incubation period, 0.1 mL of each bacterial suspension was spread on the surface of nutrient agar and incubated at 37 °C for 24 h. A single colony was placed into a test tube containing 5 mL of normal saline to produce a bacterial suspension of moderate turbidity comparable to the standard turbidity solution, approximately equivalent to 1.5 × 10^8^ CFU/mL. A sterile cotton swab was utilized to carefully transfer a portion of the bacterial suspension, which was then uniformly spread on Mueller–Hinton agar medium and incubated for 10 min. Wells with a diameter of five millimeters were created in the preceding agar layer, with three wells per plate designated for AuNPs, extract, and HAuCl_4_ solution. Agar dishes were removed, and 50 µL of AuNPs, extract, and HAuCl_4_ solution were added into each well using a micropipette. Plates were incubated at 37 °C for 18 h, after which the diameters of the inhibition zones were measured in millimeters [54].

### 3.11. Minimum Inhibitory Concentration (MIC)

Double serial dilutions (0.52–39.6 μg/mL) of AuNPs were prepared from a stock solution (10 mg/mL) in a microtiter plate utilizing Mueller–Hinton broth as the diluent. All wells, excluding the negative control wells, were inoculated with 20 μL of a bacterial suspension according to McFarland standard no. 0.5 (1.5 × 108 CFU/mL). Microtiter plates were incubated at 37 °C for 18 to 20 h. After incubation, 20 µL of resazurin dye was added to each well and incubated for additional 2 h to monitor any color changes. The sub-minimum inhibitory concentration was visually assessed in broth microdilutions as the minimal levels at which the color changed from blue to pink in the resazurin broth assay [55].

### 3.12. DPPH Free Radical Scavenging Activity

The in vitro antioxidant activity of *Eruca sativa* leaves extract, AuNPs, and vitamin C was assessed using the DPPH (2,2-diphenyl-1-picrylhydrazyl) free radical scavenging assay [56]. In brief, 2 mL of different concentrations (10, 20, 40, 80, and 100 μg/mL) were mixed with 2 mL of DPPH (mg/mL) solution in methanol. Tubes were incubated in the dark for up to 30 min. Subsequently, sample absorbance was measured at λ = 517 nm. DPPH solution without AuNPs was used as a control. Vitamin C and methanol solution were used as standard and blank, respectively. The DPPH radical scavenging activity was determined using the following equation:

DPPH scavenging activity (%) = (A0 − A1)/A0 × 100

where A0 = absorbance of the control; A1 = absorbance of the sample.

### 3.13. Statistical Analysis

All markers represented as mean ± standard deviation (SD). The differences in levels of these markers among study groups were statistically measured by F-test (ANOVA). Duncan’s test was performed to measure differences among mean levels. A *p*-value ≤ 0.05 was considered statistically significant. The data were analyzed by SPSS v. 20.0 and Microsoft Office Excel 2013 statistical software. All biological experiments were conducted in triplicates.

## 4. Conclusions

Novel gold nanoparticles (AuNPs) were synthesized using a green synthetic approach using *Eruca sativa* plant leaves aqueous extract and characterized using UV-Vis spectroscopy, FESEM, and AFM analysis, XRD, and Zeta potential analysis. The evaluation of hemolytic activity demonstrated that AuNPs exhibit favorable biocompatibility, with hemolysis rates less than 1%, suggesting no harm to red blood cells and implying their prospective safety for biomedical applications. At a high AuNPs concentration (52.8 µg/mL), an elevation in liver enzymes LDH was observed, while the lower concentration (26.4 µg/mL) induced only mild alterations in LDH. All other tested liver and kidney biomarkers, including ALT, AST, ALP, urea, and creatinine, showed no significant impact from AuNPs and therefore may support their use in biomedical applications. This protection probably reduced the damage to both hepatic and renal cells. Furthermore, the newly developed AuNPs were biologically evaluated against five different microorganisms, namely two Gram-positive (*S. aureus*, *S. pneumonia*), two Gram-negative (*P. aeruginosa*, *E. coli*), and one fungal strain (*C. albicans*). The minimum inhibitory concentrations (MIC) were measured, revealing potential activity and selectivity of AuNPs towards Gram-positive bacteria. The antioxidant profile of AuNPs and plant aqueous extract was measured using the DPPH assay and compared with vitamin C, the standard control. AuNPs were found to be superior to vitamin C, indicating their ability to act as antioxidant that may be useful for combating cellular damage caused by oxidizing agents such as free radicals. In conclusion, the green synthesized AuNPs using *Eruca sativa* aqueous leaves extract showed strong biocompatibility in vivo, and the new product comprises antimicrobial and antioxidant activity. Therefore, this study suggests that green-synthesized AuNPs may have promising applications at very low doses (in the µg/mL range) as an alternative to clinical drugs for combating antimicrobial resistance.

## Figures and Tables

**Table 1:** Hemolytic activity of AuNPs with corresponding absorbance values at 415 nm.

Sample	Nanoparticles and Controls	Absorbance (λ = 415 nm) AU ^1^
AuNPs	Gold nanoparticles	0.095
Saline	Negative control	0.087
Deionized water	Positive control	0.991

^1^ AU; arbitrary unit.

**Table 2:** Comparative mean levels of hepatic function indicator among study groups.

Parameters	Group	Mean	SD	*p*-Value
ALT (U/L)	Control	64.63 *^ns^*	4.66	*p* > 0.05
	Plant extract	58.60 *^ns^*	5.92	
	AuNPs 26.4 µg/mL	84.80 *	10.05	
	AuNPs 52.8 µg/mL	63.17 *^ns^*	5.50	
AST (U/L)	Control	140.30 *^ns^*	21.34	*p* > 0.05
	Plant extract	143.43 *^ns^*	34.84	
	AuNPs 26.4 µg/mL	156.53 *^ns^*	26.17	
	AuNPs 52.8 µg/mL	133.60 *^ns^*	51.21	
ALP (U/L)	Control	536.00 *^ns^*	173.22	*p* > 0.05
	Plant extract	583.33 *^ns^*	89.18	
	AuNPs 26.4 µg/mL	629.00 *^ns^*	8.72	
	AuNPs 52.8 µg/mL	525.67 *^ns^*	95.13	
LDH (U/L)	Control	317.33 **	56.58	*p* < 0.001
	Plant extract	434.33 **	289.83	
	AuNPs 26.4 µg/mL	203.00 **	116.05	
	AuNPs 52.8 µg/mL	1326.67 **	206.40	

Statistical significance is indicated as follows: *p* < 0.05 (*), *p* < 0.001 (**), and *ns* (non-significant); mean differences were measured using Duncan’s test [39].

**Table 3:** Comparative mean levels of renal function indicator among study groups.

Parameters	Group	Mean	SD	*p*-Value
Urea	Control	50.00 *^ns^*	14.36	*p* > 0.05
(mg/dL)	Plant extract	51.23 *^ns^*	5.55	
	AuNPs 26.4 µg/mL	55.87 *^ns^*	1.08	
	AuNPs 52.8 µg/mL	64.70 *^ns^*	5.72	
Creatinine	Control	0.73 *^ns^*	0.03	*p* > 0.05
(mg/dL)	Plant extract	0.86 *^ns^*	0.14	
	AuNPs 26.4 µg/mL	0.81 *^ns^*	0.12	
	AuNPs 52.8 µg/mL	0.82 *^ns^*	0.08	

*^ns^* Refers to non-significant; mean differences were measured using Duncan’s test [39].

**Table 4:** Inhibition zones for AuNPs, *Eruca sativa* extract, and HAuCl_4_.

Microorganism	*S. aureus*	*S. pneumoniae*	*P. aeruginosa*	*E. coli*	*C. albicans*
Inhibition zone (mm)	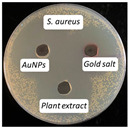	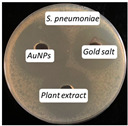	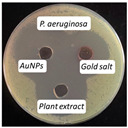	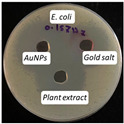	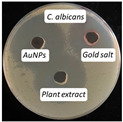
AuNPs	40	40	35	36	40
Plant Extract	37	35	35	34	35
HAuCl_4_	30	29	30	28	30

**Table 5:** Antioxidant activity (% inhibition) of biosynthesized AuNPs, plant extract, and vitamin C at different test concentrations.

Concentration (µg/mL)	AuNPs ^1^	Plant Extract ^1^	Vitamin C ^1^	*p*-Value *
100	97.22	88.56	96.29	*p* < 0.05
80	84.98	71.21	87.90	*p* < 0.05
60	63.76	51.90	58.09	*p* < 0.05
40	45.65	36.20	48.04	*p* < 0.05
20	19.27	24.31	29.21	*p* < 0.05

^1^ % Inhibition data; * Significant difference (*p* < 0.05); mean differences were calculated using Duncan test [39].

## Data Availability

All data are made available in the submitted manuscript.

## Abbreviations

The following abbreviations are used in this manuscript:

A0	Absorbance of the control
A1	Absorbance of the sample
AFM	Atomic Force Microscopy
ALP	Alkaline phosphatase
ALT	Alanine transaminase
AMR	Antimicrobial resistance
AST	Aspartate aminotransferase
AU	Arbitrary unit
AuNPs	gold nanoparticles
dl	Deciliter
DPPH	2,2-DiPhenyl-1-Picrylhydrazyl
FESEM	Field Emission Scanning Electron Microscope
FTIR	Fourier Transform Infrared Spectroscopy
HC	Hemolytic Activity
ICDD	International Centre for Diffraction Data
ICP-MS	Inductively coupled plasma-mass spectrometry
IU	International Unit
kx	Thousands of times magnification
LDH	Lactate dehydrogenase
MIC	Minimum inhibitory concentration
NC	Negative control
O	Optical density
PC	Positive control
*p*-value	Probability value
ROS	Reactive Oxygen Species
SPR	Surface Plasmon Resonance
U/L	Unit/Liter
UV-Vis	Ultraviolet–visible
XRD	X-Ray Diffraction
